# A case report of hypertrophic olivine degeneration complicated with OSAHS exacerbation: Case report and retrospective analysis

**DOI:** 10.1097/MD.0000000000032681

**Published:** 2023-01-13

**Authors:** Minxia Geng, Lulu Wang, Jiahao Xing, Shuang Li, Lulu Dong, Chao Jiang, Tianjun Wang

**Affiliations:** a Department of Neurology, Hebei General Hospital, Shijiazhuang, Hebei, China; b Graduate School of North China University of Science and Technology, Tangshan, Hebei, China; c Graduate School of Hebei Medical University, Shijiazhuang, Hebei, China; d Graduate School of Hebei North University, Zhangjiakou, Hebei, China.

**Keywords:** Guillain–Mollaret triangle (GMT), hypertrophic inferior olivary degeneration, obstructive sleep apnea hypopnea syndrome (OSAHS)

## Abstract

Hypertrophic subolivine degeneration (HOD) was destroyed by Guillain–Mollaret triangle (GMT) due to various injuries, resulting in anterograde cavity-like degeneration of the lower olive nucleus. In addition, the brain stem is related to the muscle coordination of the upper respiratory tract. Obstructive sleep apnea hypopnea syndrome may affect the respiratory center due to the injury of the brain stem. Brain stem hemorrhage damage GMT, resulting in transsynaptic neuronal degeneration. Clinical manifestations can be complex, and enhanced magnetic resonance imaging can be helpful in distinguishing them.

**Patient concerns:**

HOD is a self-limiting disease with no effective treatment and a long course of disease. Most patients can improve their symptoms after symptomatic treatment, and some patients can relieve their symptoms by themselves after 3 to 4 years.

**Diagnosis interventions:**

The limbs wobble involuntarily. His clinical symptoms and signs are consistent with HOD. Imaging with a clear primary lesion confirmed HOD. After treatment with antiepileptic drugs, the patient’s symptoms were relieved. Moreover, the patient had snoring and apnea, and respiratory sleep monitoring showed moderate obstructive sleep apnea hypopnea syndrome, which was treated with noninvasive ventilator.

**Outcomes:**

After treatment with antiepileptic drugs and noninvasive ventilator, the patient’s symptoms were significantly relieved.

**Lessons:**

HOD is a rare clinical disorder. Therefore, for similar patients, more attention should be paid to early diagnosis and treatment to avoid missed diagnosis, misdiagnosis and unnecessary intervention measures. The diagnosis can be confirmed by primary disease, clinical symptoms, and imaging based on GMT.

## 1. Introduction

Hypertrophic subolivine degeneration (HOD) is a rare transsynaptic neuronal degeneration secondary to damage to the dentate-red-olivine loop. This disease is a self-limited disease with palatal tremor as typical symptom, and the diagnosis mainly depends on imaging. Obstructive sleep apnea hypopnea syndrome (OSAHS) is a disorder characterized by symptoms such as snoring, breath-holding, drowsiness, increased urine production at night, daytime headaches, dizziness, and recurrent apnea or hypopnea during sleep. OSAHS can damage multiple systems, including cardiovascular, cerebrovascular, and metabolic systems. This paper reports the diagnosis and treatment of a HOD patient with OSAHS. It is reported below to improve clinicians’ understanding of hypohypertrophic olivine degeneration.

## 2. Case presentation

**Figure 1.**
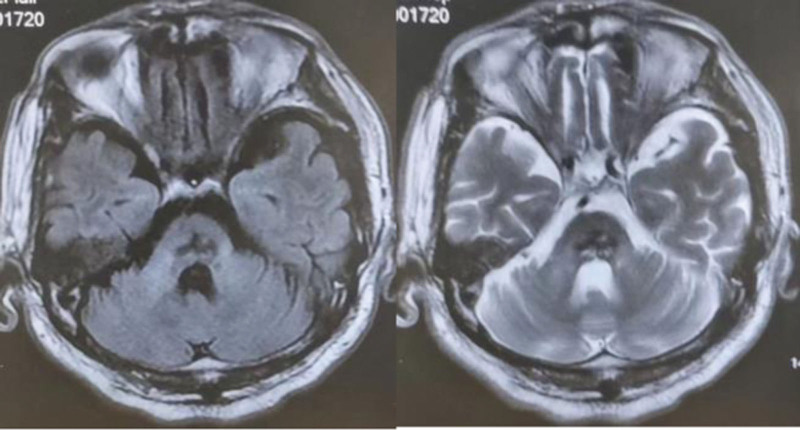
MRI image of brain stem hemorrhage at 5 months, bilateral central tegmental tract is more likely to be damaged. MRI = magnetic resonance imaging.

The patient, a 52-year-old male, was admitted to the Department of Neurology of our hospital on October 25, 2022, mainly due to involuntary limb jitter for >1 month and aggravated for 1 day. One month before admission, the patient showed involuntary limb shaking without obvious inducement, mainly in the upper limbs, at night, with no obvious pattern, accompanied by sleepiness, accompanied by dizziness, visual rotation, and double vision when both eyes were in the right and left. During this period, he was admitted to the local hospital and given conservative treatment (specific details are unknown). His family complained that the patient’s involuntary limb-shaking did not improve significantly. The involuntary vibration of the limbs was worse than before, accompanied by weakness of the lower limbs. During the course of the disease, the patient had poor diet and sleep, dry stools, and normal urination. The patient had no symptoms such as fever, cough, sore throat, and diarrhea before onset. He had a 1-year history of hypertension with a maximum of 160/115 mm Hg. He regularly took antihypertensive drugs and controlled them at 140/110 mm Hg. He had a history of brain stem hemorrhage 5 months before admission, underwent tracheotomy, left a speech problem, could not eat on his own (indwelling gastric tube), was bedridden, and could not stand. Denial of diabetes, coronary heart disease, and other medical history; no history of tuberculosis and other infectious diseases; no history of trauma; denial of allergies to drugs, food, etc. Physical examination: body temperature 36.2ºC, heart rate 66 times/min, breathing 18 times/min, blood pressure 131/91 mm Hg. Double lungs breathing sound coarse, can hear phlegm sound, regular heart rhythm, and each valve auscultation area did not hear and murmur. Abdomen is flat and soft, no obvious tenderness or rebound pain. Lethargy, slurred speech, slow reaction, uncooperative physical examination, double pupils large and equal round, sensitive light reflex, both eyes can move in all directions, left and right visual objects in pairs, horizontal and vertical eye tremors, bilateral soft palate involuntary tremor, uvula centered, and disappearance of pharyngeal reflex. Bilateral frontal line symmetry, nasolabial groove symmetry, indicating the mouth angle is not biased, extended tongue center, and tongue muscle fibrillation. Head-turning and shoulder-hunching are strong, bilateral limb muscle tone is slightly low, double upper limb muscle strength level 5, double lower limb muscle strength level 5−. Involuntary tremor of limbs, ataxia movement: bilateral rotation test, finger nose test, heel-knee-shin test and other physical examination did not cooperate, and pathological signs and meningeal stimulation signs were negative. Auxiliary examination: after admission, blood gas analysis was improved for 2 times (Table 1), complete abnormalities were found in 2 emergencies, routine abnormalities were found in 2 urinalysis, and video electroencephalogram (EEG) (1 day after admission) indicated abnormal EEG. Onset: Dozens of episodes were detected. EEG: No epileptic discharge was observed, and kinetic artifacts were observed. Intermittent period: diffuse slow wave activity increased in each lead. Episode: an episodic event. Reexamination of EEG (7 days after admission): abnormal EEG, EEG topographic map, multiple medium amplitude θ-wave unit δ-wave in each lead, fast wave: a small amount of low amplitude β-wave. Respiratory sleep monitoring (7 days after admission): moderate OSAHS. Imaging examination: cranial magnetic resonance angiography (MRA) (at the local hospital 1 month before admission): formation of pons softening lesion and abnormal signal shadow in the anterior medulla oblongata. Craniocerebral MRA: cerebral atherosclerotic changes (M2 segment of both middle cerebral arteries, right posterior cerebral artery). Head computed tomography scan (15 days before admission): left basal ganglia ischemic lesion was considered; brain atrophy. Head computed tomography + 3D reconstruction indicated (1 day after admission): bilateral lacunar cerebral infarction in basal ganglia area, small softening lesion in basal ganglia area and left pons. Cranial diffusion-weighted imaging (DWI) + MRA: No obvious abnormally high signal was observed on cranial DWI imaging. The right anterior cerebral artery A1 segment and the right middle cerebral artery M1 segment were localized stenosis. Head magnetic resonance imaging (MRI) + susceptibility weighted imaging (Fig. 1): patchy abnormal signal shadow in the pontine, considering hemorrhagic malacia foci; multiple small softening foci in bilateral basal ganglia and left thalamus. Treatment process: the patient had an acute onset and a history of hypertension and cerebral hemorrhage. This time, the patient had bilateral involuntary tremor of the soft palate, tongue muscle fibrillation, binocular nystagmus, diplopia, involuntary shaking of the limbs and disturbance of consciousness. Imaging suggested that the lesion was located in the brain stem, so it was diagnosed as a degenerative disease of the nervous system: hyperplasia of inferior olivine degeneration. Symptomatic treatment after admission, antiepileptic drugs valproate sustained-release tablets and levetiracetam were given to control limb vibration, but the effect of limb shaking was not good on the 3rd day after admission. After topiramate 25 mg was added once every 12 hours, the symptoms were relieved, levetiracetam was reduced, benzoxol hydrochloride 2 mg was added once every 24 hours, and the spirit was significantly improved. On the 12th day after admission, the patient still had intermittent involuntary shaking at night. After the addition of clonazepam tablets before going to bed, the shaking during the sleep period was significantly reduced, which supported hypertrophic olivary degeneration. And give antihypertensive, open the body to wake up the brain, protect the stomach, promote collateral circulation and other treatments. Supplementary diagnosis: OSAHS. The patient’s family members purchased portable ventilators by themselves and were given noninvasive assisted ventilation. After treatment, oxygen partial pressure increased significantly and dizziness and other symptoms were relieved.

**Table 1:** Blood gas analysis of the patient after admission.

Time	pH	PaCO2 (mm Hg)	PaO2 (mm Hg)	SpO2 (%)	K^+^ (mmol/L)	Ca^2+^ (mmol/L)	Glu (mmol/L)
3 d after admission	7.4	47.13	60.37	89.95	3.31	1.03	7.54
10 d after admission	7.37	43.45	72.02	95.43	3.37	1.03	8.31

## 3. Discussion

**Figure 2.**
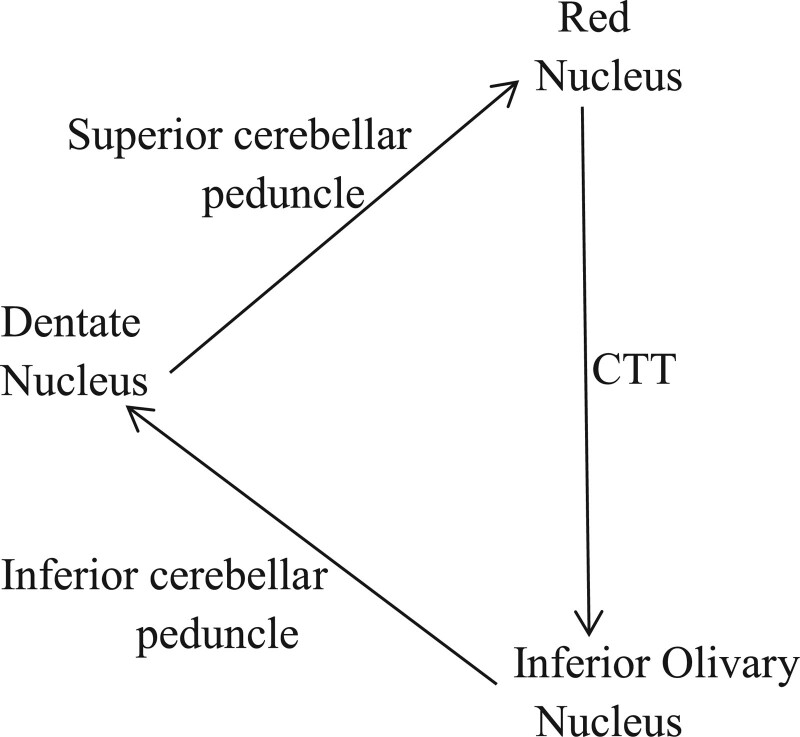
Guillain–Mollaret triangle.

HOD, also known as hypertrophic olivine degeneration, is primarily a destructive lesion of the brain stem or cerebellum secondary to the Guillain–Mollaret triangle (GMT).^[1]^ In 1931, Guillain et al^[2]^ pointed out the anatomical region of this triangle and described GMT in detail, that is, 1 side of the midbrain red nucleus projected from the contralateral cerebellar dentate nucleus through the superior leg of the cerebellar, and the ipsilateral submedulla olivary nucleus projected from the central tegmental tract to the midbrain red nucleus. Then, it is projected to the contralateral cerebellar cortex and the dentate nucleus of the cerebellar through the lower leg of the cerebellar to form a loop (Fig. 2). Guillian–Mollaret anatomy damage to any point on the triangular loop will cause degeneration of the lower olive nucleus. Therefore, the HOD may be caused by cerebral hemorrhage, cerebral infarction, tumor, inflammation, trauma, degeneration, and brain stem cavernoma. It is important to understand the composition of GMT to understand how it affects anterograde cavity-like degeneration of the olivary nucleus. In this case, the patient had a history of brain stem hemorrhage, blocking the GMT pathway and triggering HOD.

The pathogenesis of HOD is unclear and may be related to disinhibition of the lower olivary nucleus. When the upstream afferent nerve is inhibited, hypertrophy degeneration occurs in the lower olive nucleus after overexcitation. HOD pathological changes could be divided into 6 stages according to Goto et al^[3]^: on day 1, there was no change in the lower olive nucleus; about 2 to 7 days or a long time, the white matter olive sac will gradually degenerate; at about 3 weeks, the lower olivary nucleus showed mild hypertrophy, mitochondria increased significantly, but no glial reaction; at about 8.5 months, the neurons and astrocytes were hypertrophic, and the inferior olivary nucleus was larger than that before; after 9.5 months, the inferior olivary nucleus showed pseudohyperplasia, in which some neurons were completely dissolved with astrocytes; and after 3 to 4 years, the neurons basically disappeared and the inferior olivary nucleus decreased significantly. HOD is mainly examined by imaging technique MRI. It mainly reflects high or slightly high signal on T2WI, low or equal signal on T1WI, fluid attenuated inversion recovery (FLAIR) or slightly high signal on FLAIR, DWI or slightly high signal on ADC, susceptibility weighted imaging or slightly high signal on lower olive nucleus. The high signal on T2WI was persistent. There are usually 3 phases on MRI^[4]^: phase 1 usually occurs 1 month after the onset of the disease, when the lower olivary nucleus shows high signal on T2WI, with enhanced signal on FLAIR and normal volume. Stage 2 usually occurs 4 to 6 months after the onset of the disease, with high T2 signal and enlargement of the lower olivary nucleus. Stage 3, still maintains T2 hypersignal and persists, usually within 3 to 4 years to normalize or reduce the volume of the lower olive nucleus. Therefore, HOD is liable to be misdiagnosed and missed clinically. Timely differential diagnosis should be made to exclude other diseases for timely treatment.

HOD lesions can be either be ipsilateral, contralateral, or bilateral. If the lesion is located in the brain stem, an ipsilateral HOD is produced. If the lesion is located in the cerebellum, it results in a contralateral HOD; if the lesions involve these structures at the same time, bilateral HOD may result. In addition, it was found that gene mutations could also lead to HOD, such as POLG gene and SURF1 gene, which could lead to mitochondrial defects and then to HOD.^[5]^ Therefore, this is attributed to mitochondrial disorders. Palatal myoclonus is the most typical clinical feature, which is characterized by myoclonus contractions with a rhythm of 1 to 3 Hz, sometimes involving the throat, tongue and face, and movement disorders such as nystagmus, double vision, eye muscle paralysis, eye myoclonus, Holmes tremor and cerebellar ataxia may occur, and some of the symptoms can be self-relieving after 3 to 4 years.^[6]^ The patient presented with limbs clonus, inability to stand, bilateral tremors of the soft palate, lingual muscle fibrillation, disappearance of pharyngeal reflex, mandibular jitter, binocular vision, and binocular nystagmus.

The patient presented with drowsiness, lethargy, snoring, apnea, dizziness, visual rotation, and shaking during sleep. The respiratory sleep monitoring showed OSAHS. We considered that the previous bleeding in the brain stem may have affected the respiratory center, and the coordination between the brain stem and the muscles of the upper respiratory tract was related. As the disease progresses, the brain stem hemorrhage triggers the HOD, with subsequent exacerbation of the obstructive sleep apnea hypoventilation syndrome. Analysis of the HOD caused by brain stem hemorrhage in this patient may aggravate OSAHS.

OSAHS is a systemic disease that causes abnormal sleep disorders due to the repeated awakening of the cerebral cortex at night and the disruption of the structure and rhythm of sleep, which is manifested as daytime fatigue and lethargy, etc. Physical examination may show the thickness of uvula, etc, and may cause serious damage to cognitive function and behavioral abnormalities. Repeated nighttime apnea and hypopnea can decrease PaO2, increase PaCO2, increase sympathetic nerve excitability, and lead to decreased antioxidant capacity, which may cause or aggravate cerebrovascular diseases. The incidence of OSAHS is mainly related to the muscle discoordination of the respiratory tract. Zha et al^[7]^ studied the upper airway of patients with OSAHS through MRI, and believed that with the aggravation of OSAHS patients, the cross-sectional area of the upper airway decreases, and the stenosis degree is positively correlated with the severity of OSAHS, and the severity can be graded by AHI. The patient was in moderate condition, suffering from typical snoring with apnea, hypertension, and cerebrovascular diseases. After using a noninvasive ventilator, apnea improved significantly.

According to the literature, there is no effective treatment for HOD at present, mainly symptomatic treatment to relieve symptoms. Arican et al^[8]^ found that sodium valproate and clonazepam, etc could reduce HOD symptoms through clinical treatment. In this case, the patient received levetiracetam and other treatments, and also had a certain effect. When HOD with OSAHS is encountered clinically, symptoms may be relieved by continuous positive airway pressure. To sum up, it is not clear whether there is any further association between HOD and OSAHS. We will continue to follow up and observe the progression of the disease in this patient. We report the cases of this rare phenomenon in order to enrich the clinical connotation of this disease and avoid the delay of treatment due to missed diagnosis and misdiagnosis.

## Acknowledgments

The authors would like to thank Tianjun-Wang for her assistance in writing this manuscript.

## Author contributions

**Conceptualization:** Minxia Geng.

Data curation: Shuang Li.

Formal analysis: Tianjun Wang.

Funding acquisition: Lulu Wang.

Investigation: Minxia Geng.

Methodology: Minxia Geng, Lulu Wang, Jiahao Xing, Lulu Dong.

Project administration: Shuang Li.

Resources: Jiahao Xing.

Software: Jiahao Xing, Lulu Dong.

Supervision: Lulu Wang, Tianjun Wang.

Validation: Shuang Li, Chao Jiang.

Visualization: Chao Jiang.

Writing – original draft: Minxia Geng.

**Writing – review & editing:** Minxia Geng, Lulu Wang, Tianjun Wang.
